# Beliefs about coronavirus: Relationship with magical thinking and adherence to self-isolation regimen

**DOI:** 10.1192/j.eurpsy.2021.754

**Published:** 2021-08-13

**Authors:** S. Kumchenko, E. Rasskazova, A. Tkhostov

**Affiliations:** 1 Clinical Psychology, Moscow State University, Moscow, Russian Federation; 2 Department Of Neuro- And Pathopsychology, Faculty Of Psychology, Lomonosov Moscow State University, Moscow, Russian Federation

**Keywords:** infodemic, coronavirus, magical thinking

## Abstract

**Introduction:**

Pandemic is accompanied by “infodemic” that is related to higher anxiety (Moghanibashi-Mansourieh, 2020; Roy et al., 2020; Huang, Zhao, 2020). We suggest that indefinite and stressful situation of pandemic provoke magical thinking leading to lower adherence with recommendations for self-isolation.

**Objectives:**

The aim was to reveal the structure of beliefs about reasons, manifestation and consequences of coronavirus and their relationship with magical thinking, anxiety and COVID-19-related behaviour.

**Methods:**

In April 2020 (2-3 weeks of self-isolation regimen) 402 adults aged 18-64 years old filled checklist including beliefs about pandemic (based on the model of Leventhal et al., 2003), Magical Ideation Scale Eckblad, Chapman, 1983) as well as scales measuring anxiety and protective behaviour in pandemic and monitoring of information about coronavirus (Tkhostov, Rasskazova, 2020).

**Results:**

Factor analysis revealed three groups of radical beliefs about coronavirus (48.6% of variance, Cronbach’s alphas .62-.75). Belief about the particular meaning of coronavirus was associated with the magical thinking (r=.21), less anxiety about infection (r=-.19) and poorer adherence to self-isolation (r=-.26). Belief in the negligence as a cause of coronavirus was more typical for those with better adherence (r=.18) while catastrophic beliefs about the consequences of pandemic were related to frequent monitoring of the information about the pandemic (r=.24), and anxiety regarding future negative consequences of the pandemic (r=.46).

**Conclusions:**

Dysfunctional beliefs about coronavirus could be a factor of poorer adherence related to magical thinking and could be addressed in psychological interventions. Research is supported by the Russian Foundation for Basic Research, project No. 20-04-60072.

